# Accuracy and consequences of same-day, invasive lung cancer workup – a retrospective study in patients treated with surgical resection

**DOI:** 10.3402/ecrj.v3.32590

**Published:** 2016-11-30

**Authors:** Kirsten Riis Madsen, Asbjørn Høegholm, Uffe Bodtger

**Affiliations:** 1 Department of Internal and Respiratory Medicine, Naestved Hospital, Naestved, Denmark; 2 Institute of Regional Health Research, University of Southern Denmark, Odense, Denmark

**Keywords:** lung cancer, diagnosis, bronchoscopy, EBUS, TTNAB

## Abstract

**Background:**

Though widely used, little is known about accuracy and efficacy of same-day, invasive workup of suspected lung cancer.

**Objective:**

To evaluate the accuracy and efficacy of same-day, invasive lung cancer workup (diagnosis and mediastinal staging), and to identify differences between patients without (Group A) or with (Group B) need for resampling.

**Methods:**

A retrospective study was performed on all consecutive patients referred for surgical treatment for localised lung cancer after invasive diagnostic and staging workup at our unit. Data were extracted from electronic medical files. Surgical specimens served as gold standard for correct diagnosis and stage.

**Results:**

A total of 129 patients (peripheral lesion: 84%; mediastinal staging: 97%) were included. After same-day, invasive workup, 71% had no need for further invasive workup (Group A), while 29% had (Group B). Group A differed significantly from Group B in fewer invasive tests, fewer days from referral to surgery, and lower pneumothorax incidence, while no differences were observed in diagnostic accuracy, cancer subtype, tumour size, tumour stage, peripheral lesion, nodal involvement, gender, or presence of chronic obstructive pulmonary disease. Tumour located in right upper lobe was associated with need for resampling.

**Discussion:**

Our retrospective study suggests that same-day, invasive workup for lung cancer is safe, accurate, and efficacious in reducing time to therapy, even in patients with small lesions and low tumour burden.

Primary lung cancer is the second most incident cancer in both men and women, and is worldwide the most common cause of cancer-related deaths (1, 2). Correct diagnosis and staging is pivotal for optimal treatment as treatment modality and prognosis differ significantly according to tumour type and dissemination, staged according to the Union for International Cancer Control (UICC) tumour–lymph node–metastasis (TNM) classification system (3, 4). Furthermore, time from diagnosis to surgical resection increased from 1995 to 2005 in the United States (5); thus, there is a need for initiatives to reduce this avoidable delay.

Surgical resection is offered for patients with localised disease, documented by radiographic and endoscopic staging (3). Tissue sampling for cytology or histology is mandatory, and has generally high specificity regardless of tumour type or stage, whereas diagnostic sensitivity and negative predictive value decrease with lesion size (6). Thus, diagnostic workup of patients eligible for curative treatment is associated with the lowest diagnostic sensitivity (6, 7). No single, invasive procedure alone can diagnose and stage lung cancer in patients with radiologically evident localised disease (3, 6, 7). The evidence of the combined diagnostic efficacy of the multiple invasive tests performed in a single session is sparse, whereas very solid for each individual diagnostic procedure (6, 8).

The focus of this retrospective study was to investigate the accuracy and safety of a combined diagnostic approach in patients with surgically resected non-small cell lung cancer (NSCLC) with surgical specimens being the gold standard of diagnosis and stage.

## Methods

### Design

A retrospective, non-interventional, data collection study was performed on the basis of electronic medical data charts.

### Major endpoint

Accuracy of lung cancer workup: % correct cytopathological diagnosis and tumour and nodal (T–N) stage decided at multidisciplinary team (MDT) conference compared to Gold Standard (postsurgical histopathological diagnosis and T–N stage) including intergroup differences (Group A: patients with completed diagnosis and staging after a single visit; Group B: patients requiring 2 visits).

### Minor endpoint

Intergroup differences in time from referral to diagnosis are surgery, demographic data, imaging data, tobacco pack years, airflow obstruction, number and nature of procedures performed, comorbidity, and performance status.

### Definition

A ‘conclusive workup (diagnosis and staging)’ was obtained when a determinate TNM stage was decided at the MDT conference.

### Patients

The study included all patients from our institution (Department of Pulmonology, Naestved Hospital, Denmark) who underwent invasive diagnostic workup and were later referred for intended curative surgery for lung cancer.

The study period was 1 January 2009 to 31 December 2011. A total of 937 patients were diagnosed with lung cancer in our unit during the study period.

### Diagnostic centre

All patients were initially referred for diagnosis and staging of suspected lung cancer to our institution where experienced pulmonologists, radiologists, cytopathologists, and oncologists are available with rapid access to computerised tomography (CT) and positron emission tomography with low-dose CT (PET-CT). Inter-hospital agreements provide access to surgical procedures at the Department of Thoracic Surgery at University Hospitals in either Odense or Copenhagen (Denmark). In our unit, we perform bronchoscopies, endobronchial ultrasound (EBUS), and/or endoscopic ultrasound (EUS) as outpatient procedures in conscious sedation (intravenous midazolam and fentanyl). Furthermore, we perform ultrasound-, X-ray-, or CT-guided transthoracic needle aspiration biopsies (TTNABs); pleurocenteses; and non-endoscopic, non-thoracic, ultrasound-guided biopsies (fine-needle aspiration [FNA] biopsies from liver, spleen, skin tumours, bone lesions, or superficial lymph nodes) in local analgesia.

### Diagnostic workup

Briefly, only experienced pulmonologists were involved in lung cancer workup. Visit 1 was defined as the first visit with invasive tissue sampling. At our unit, visit 1 included medical history (including Charlson's index of comorbidity), physical examination [including ECOG (Eastern Cooperative Oncology Group) performance), spirometry, tissue sampling, and lastly informing patient of the preliminary results and suspicion (including macroscopic findings).

### Pre-operative staging

Conclusive results were presented at the local MDT conference with attendance of local pulmonologist, radiologist, cytopathologist, oncologist, and a thoracic surgeon (later presented by video transmission). TNM stage was assessed (9), and patients were referred to relevant therapy according to cytology, TNM, and performance status (10).

The post-surgical diagnosis and stage was considered the gold standard of T–N stage.

### Ethics

This study was a retrospective observational study aimed to examine the quality of previously performed procedures. There was no randomisation, intervention, or study-specific collection of biological material, and thus the study do not fall under the jurisdiction of ethics committee system. Data collection was approved by The Danish Data Protection Agency.

### Data analysis

All data were collected from medical records. Each visit to Naestved Hospital that included an invasive procedure (excluding blood sampling) was noted and counted. All statistical analyses were performed using commercially available software (SPSS version 20, IBM, USA). Discrete data were presented as median (range), and binary data as percentage. Differences were examined with non-parametric testing (Mann–Whitney *U*-test and chi-square test). Significance was reached when *p* was less than 0.05.

## Results

### Patients

A total of 133 patients were referred for surgery (14% of the 937 patients diagnosed with lung cancer during the study period). Four patients were excluded: pre-operative staging (after neo-adjuvant chemotherapy) performed by thoracic surgeons (*n*=1), or no invasive diagnostic procedures performed (small, peripheral lesions, and radiographically normal mediastinal lymph nodes; *n*=3).

Table 1 depicts data on the 129 included patients (female*=*64; median age=69 [range 19–84] years). We found no gender- or age-related differences concerning any variable (data not shown).

**Table 1:** Demographic and basic clinical data on patients with completed workup (Group A) versus incomplete workup (Group B) after multiple invasive tests at a single visit

	≤1 visit, *n*=91 (Group A)	≥2 visit, *n*=38 (Group B)	*p*^a^
Female, *n* (%)	49 (54%)	15 (40%)	n.s.
Age, median (SD)	68 (37–84)	69 (19–79)	n.s.
Asymptomatic, *n* (%)	36 (40%)	14 (37%)	n.s.
Formerly cancer afflicted, *n* (%)	23 (25%)	3 (8%)	0.03
Pack years, median (range)	40 (0–80)	40 (0–80)	n.s.
Smoking status			n.s.
Smoker, *n* (%)	47 (52%)	17 (45%)	
Former smoker, *n* (%)	40 (44%)	18 (47%)	
Never smoker, *n* (%)	4 (4%)	3 (8%)	
Chronic obstructive lung disease	46 (64%)	14 (52%)	n.s.
FEV_1%_ predicted, median (range)	77 (36–145)	82 (23–133)	n.s.
ECOG performance, median (range)	1 (0–1)	0 (0–2)	
Charlson's index, median (range)	1 (0–8)	1 (0–4)	n.s.
CT, *n* (%)	87 (96%)	37 (97%)	n.s.
PET-CT, *n* (%)	89 (98%)	37 (97%)	n.s.
PET+ mediastinal lymph nodes, *n* (%)	15 (17%)	8 (21%)	n.s.
Tumour mean diameter, median (range)	30 (10–100)	38 (14–83)	0.09
Tumour size ≤20 mm, *n* (%),	22 (24%)	7 (18%)	n.s.
Peripheral lesion	75 (82%)	33 (87%)	n.s.
Lobe affected			0.038
Right upper	21 (23%)	17 (45%)	
Right middle	3 (3%)	2 (5%)	
Right lower	23 (25%)	4 (11%)	
Left upper	28 (31%)	6 (16%)	
Left lower	16 (17%)	9 (24%)	

a Categorical variables: chi-square or Fisher's test; discrete variables: Mann–Whitney *U*-test; n.s: non-significant.

### Lung cancer workup

Definite diagnosis and stage as decided at the MDT conference was achieved in 91 patients (71%) after a single visit. At visit 1, patients went through a bronchoscopy (97%), EBUS (98%), and TTNAB (78%), and additionally 16 patients (12%) had either a EUS-FNA of the left adrenal gland (*n*=12) or an ultrasound-guided FNA of liver lesion (*n*=3) or cervical lymph node (*n*=1) in our endoscopy unit.

Resampling was needed in 38 patients (29%) because of inconclusive diagnosis (*n*=23; 18%), conclusive diagnosis but EBUS not representative (*n*=9; 7%), both (*n*=1; 1%), no EBUS at visit 1 (*n*=2; 2%), EBUS sample adequate and benign from PET-positive node (*n*=2; 2%), and abnormal cells in N2 lymph node (*n*=1; 1%): re-bronchoscopy (once: *n*=12 patients; twice: *n*=1); re-EBUS (once: *n*=17 patients), and TTNAB (once: *n*=14; twice: *n*=2).

Workup included both a contrast-enhanced CT and a PET-CT in most patients (*n*=122; 97%). Contrast-enhanced CT alone (*n*=3) or PET-CT alone (*n*=4) was not associated with the need for resampling (Table 2). PET-scan resulted in additionally eight diagnostic procedures (Group A: *n*=6 [6%]; Group B: *n*=2 [6%]): mammography (*n*=2), gastro-duodenoscopy (*n*=2), sigmoidoscopy (*n*=3), and brain MR (*n*=1).

**Table 2:** Differences in radiographic findings, number of invasive tests and visits, prevalence of invasive tests, and mortality between groups

	≤1 visit, *n*=91(Group A)	≥2 visit, *n*=38(Group B)	*p*^a^
Days from referral to final diagnosis (MDT), median (range) [10–90 percentile]	16 (2–48)[9–27]	29 (12–197)[13–46]	<0.00001
Days from referral to surgery, median (range) [10–90 percentile]	41 (14–127)[28–66]	49 (21–246)[28–77]	<0.005
Bronchoscopy, *n* (%)	87 (96%)	37 (97%)	n.s.
Diagnostic yield	17%	13%	n.s.
EBUS, *n* (%),	88 (97%)	37 (97%)	n.s.
Diagnostic yield	14%	8%	n.s.
TTNAB, *n* (%)	70 (77%)	31 (81%)	n.s.
Diagnostic yield	92%	84%	n.s.
Total number of invasive test, median (range)	3 (1–5)	4 (2–7)	<0.00001
TRIO at day 1	67 (74%)	25 (66%)	n.s.
Futile TTNAB, *n* (%)	9 (10%)	1 (3%)	n.s.
Pre-surgical cytopathological diagnosis, *n* (%)	86 (95%)	34 (90%)	n.s.
Immunohistochemistry, *n* (%)			n.s.
Performed	54 (59%)	23 (61%)	
Microscopy conclusive	12 (13%)	6 (16%)	
Not enough material	25 (28%)	9 (24%)	

a Categorical variables: chi-square or Fisher's test; discrete variables: Mann–Whitney *U*-test; n.s: non-significant.

Having a tumour in the right upper lobe was associated with need for resampling (*p*=0.014). We found no significant association between tumour localisation in right versus left lung nor in upper lobes versus middle/lower lobes and need for resampling.

Table 3 shows incidence and spectrum of complications. There were no serious adverse events to workup.

**Table 3:** Demographic and basic clinical data on patients with completed workup (Group A) versus incomplete workup (Group B) after multiple invasive tests at a single visit

	≤1 visit, *n*=91 (Group A)	≥2 visit, *n*=38 (Group B)	*p*^a^
Visit 1			
Complication, *n* (%)	19 (21%)	7 (18%)	n.s.
Pneumothorax, no drain, no admission, *n* (%)	4 (4%)	1 (3%)	
Pneumothorax, no drain, admission, *n* (%)	5 (6%)	1 (3%)	
Pneumothorax, drain, no admission, *n* (%)	0 (0)	1 (3%)	
Pneumothorax, drain, admission, *n* (%)	7 (8%)	3 (8%)	
Other,^b^ *n* (%)	3 (3%)	1 (3%)	
Complication needing admission, *n* (%)	12 (13%)	4 (11%)	n.s.
Days in hospital, median (range)	0 (0–5)	0 (0–2)	n.s.
Pneumothorax, any, *n* (%)	14 (15%)	8 (21%)	n.s.
All visits			
Complication needing admission, *n* (%)	12 (13%)	7 (18%)	n.s.
Pneumothorax, any, *n* (%)	14 (15%)	13 (34%)	0.017

a Categorical variables: chi-square or Fisher's test; discrete variables: Mann–Whitney *U*-test

b pain, confusion, angina, and vasovagal hypotension; n.s: non-significant.

### Diagnosis and stage

Pre-operatively, a total of 120 (93%) patients were diagnosed with primary lung cancer: adenocarcinoma (*n*=64; 50%), squamous cell carcinoma (*n*=31; 24%), NSCLC/Carcinoma not otherwise specified (NOS; *n*=17; 13%), carcinoid (*n*=4; 3%), and neuroendocrine non-carcinoid tumours (*n*=4; 3%) without any significant differences between Group A versus B. The remaining nine patients (7%) had persistent and PET-positive lesions without conclusive cytopathological diagnosis despite targeted workup (Table 2). Chronic inflammation, and not malignancy, was found after lobectomy in one of these undiagnosed patients (Group B). Table 4 depicts differences in pre- and post-operative tumour (T) and nodal (N) stage.

**Table 4:** Differences in pre- and post-operative diagnosis and stage between groups

	Pre-operative findings			Post-operative findings		
		
	≤1 visit, *n*=91 (Group A)	≥2 visit, *n*=38 (Group B)	*p*^a^	≤1 visit, *n*=91 (Group A)	≥2 visit, *n*=38 (Group B)	*p*^a^
Diagnosis			n.s.			n.s.
Adenocarcinoma	45 (50%)	19 (50%)		52 (57%)	22 (60%)	
Squamous cell carcinoma	23 (25%)	8 (21%)		26 (29%)	10 (26%)	
Carcinoma NOS	14 (15%)	3 (8%)		3 (3%)	0 (0%)	
Neuroendocrine carcinoma^b^	1 (1%)	3 (8%)		7 (7%)	4 (11%)	
Carcinoid tumour	3 (3%)	1 (3%)		3 (3%)	1 (3%)	
Non-malignant diagnosis	5 (6%)	4 (11%)		0	1 (3%)	
Stage					(benign tumour excluded)	
T1a	19 (21%)	5 (13%)	n.s.	11 (12%)	2 (5%)	n.s.
T1b	16 (18%)	5 (13%)		13 (14%)	3 (8%)	
T2a	34 (37%)	9 (24%)		44 (48%)	18 (47%)	
T2b	12 (13%)	10 (26%)		12 (13%)	4 (11%)	
T3	8 (9%)	6 (16%)		10 (11%)	9 (24%)	
T4	2 (2%)	3 (8%)		1 (1%)	1 (3%)	
N0	81 (89%)	35 (92%)	n.s.	67 (74%)	33 (87%)	n.s.
N1	9 (10%)	3 (8%)		13 (14%)	2 (5%)	
N2	1 (1%)^c^	0 (0%)		11 (12%)	2 (5%)	
Upstaged	–	–	–	18 (20%)	3 (9%)	n.s.
Upstaged to N2				9 (10%)	2 (5%)	n.s.
Recurrence within 2 years				23 (25%)	6 (16%)	n.s.
Mortality, 30-days, *n* (%)	–	–	–	2 (2%)	1 (3%)	n.s.
Mortality 6-month, *n* (%)				6 (7%)	4 (11%)	n.s.
Mortality 12-month, *n* (%)				15 (17%)	5 (13%)	n.s.

NOS, not otherwise specified.

a Chi-square or Fisher's test;

b except carcinoid;

c a patient with CT-normal but FDG-positive lymph node station 5; n.s: non-significant.

Totally, 99 patients had a matching pre-and post-surgical diagnosis (Group A: *n*=70 [77%] vs. Group B: *n*=29 [76%]; *p*>0.5). In the remaining patients, most disagreements were observed in patients with a pre-surgical diagnosis of carcinoma NOS or no cancer: adenocarcinoma (*n*=14; 47%) and squamous cell carcinoma (*n*=5; 17%). Only one was found to have a metastasis from an extrapulmonary malignancy (Group A): a young woman with no PET-positive extra-thoracic lesions, and a pre-surgical diagnosis of large cell carcinoma indistinguishable from choriocarcinoma metastasis. Histopathological examination of the resected tumour confirmed the latter.

Table 2 shows that conclusive workup after a single visit was associated with significantly shorter interval from referral to surgery. Delayed surgery was observed in five patients because of cardiac comorbidity, trauma, and patients’ reluctance to proceed to surgery.

Median tumour size and number of patients with tumour size ≤20 mm did not differ between groups (Table 2). Comparing patients with tumour size ≤20 mm versus >20 mm, we observed significantly fewer invasive tests (median 3 [1–4] vs. 3 [1–7], *p<*0.05; mean 2.8 [SD 0.9] vs. 3.4 [SD 1.0]) but no difference in median time to surgery (40 [28–95] vs. 43 [31–70] days, *p=*0.6).

### Mortality

Overall mortality 12 months after MDT conference was 16% (*n*=20). One patient in Group A (post-surgical stage IIbN0M0 squamous cell carcinoma in left lower lobe) died within the first 48 h after surgery. Table 4 shows that mortality at 1, 6, or 12 months did not differ significantly between groups.

## Discussion

Bias is unavoidable in retrospective studies, and especially confounding-by-indication is most likely present in our retrospective study on everyday practice. Yet, this study is the first to actually address the efficacy of the widely used same-day, invasive workup of lung cancer workup, aiming at reducing the number of patients’ visits. We found that most patients were successfully diagnosed and staged after a single visit, implying fewer invasive tests and a reduced time delay to surgery, without negative impact on diagnostic accuracy or mortality. Tissue samples were obtained by endoscopic and/or transthoracic FNA biopsies according to individualised plans based on findings at contrast-enhanced CT and/or PET-CT (low-dose) scan(s). We reported only surgically resected cases as the gold standard of lung cancer workup is absent in non-resected cases. All but one patient had a malignant diagnosis, and 74% was diagnosed and staged after a single visit with tissue sampling.

In our study, we found that tumour location in right upper lobe – but neither tumour size or peripheral location, concomitant chronic obstructive pulmonary disease, low physical performance, nor PET-positive mediastinal lymph nodes – was associated with diagnostic failure (Group B; Table 1). The impact of lobar tumour location on sensitivity or negative predictive values has been sparsely investigated. A bronchoscopic study found the lowest yield when the lesion was in the most apical or basal segment (11), and a TTNAB study found the numerically lowest yield in the right upper lobe (12). The background is not known, but we speculate that the thoracic anatomy of the apical thorax restricts tumour access because of, for example, lower rib–rib distance and larger distance from skin to pleura compared to more distally located lesions. As the right upper lobe is smaller than the left, a larger proportion is located in this difficult area.

No single technique allows sufficient tissue sampling for diagnosis and staging every case of suspected lung cancer, as size, localisation, and numbers of both primary and metastatic lesions as well as patients’ performance vary significantly (4, 6, 13). Endoscopic ultrasonographic tissue sampling is considered the most cost-effective modalities, but cannot reach peripheral stage I or II lung cancer (14). The diagnostic yield of each tissue sampling technique is well documented (4, 6), but to our best knowledge, this is the first study to investigate the composite results of multiple techniques.

Our study addresses a method to reduce time delay from referral for cancer workup to treatment (15, 16). The availability of more efficient equipment is counterweighted by an increasing demand for correct diagnosis at the molecular levels in order to provide personalised treatment (3, 17). The time-consuming pre-operative workup by PET-CT, endoscopic mediastinal staging, and prediction of expected post-surgical physiology has resulted in a decrease in lung resections from 2000 to 2007 in Denmark (18). An American report showed that waiting times for surgery increased from 1995 to 2005 for the eight most common cancers but with a shorter delay when diagnosis and surgery were performed at the same hospital (5). In Denmark, lung resection is performed at four departments of thoracic surgery but lung cancer workup in 14 departments of pulmonology: the proportion of lung cancer resections performed within 14 days from referral increased from 69% to 87% from 2000 to 2007, mainly because of implementation of integrated, cross-sectional national guidelines for lung cancer workup, and reduction from 40 to 14 diagnostic departments and from 7 to 4 surgical departments (18). At our unit, we have implemented initiatives to reduce diagnostic delay: all diagnostic workup is performed by pulmonologists skilled in thoracic endoscopy (bronchoscopy, EUS, EBUS) and ultrasound-guided tissue sampling from pleura, liver, spleen, superficial lymph nodes, subcutis, and breasts (19–21). Additionally, bi-weekly MDT conferences facilitate rapid referral to oncology or surgery (5, 16, 18, 22).

Furthermore, our data show that we follow the ACCP and BTS guidelines recommendation of early mediastinal staging before surgery (3, 10). A recent US report found that this was performed only in 21% of patients (23, 24). Mediastinal workup may not be required in patients with stage Ia and normal lymph nodes at CT, in which a false-negative rate of 10% is considered acceptable according to ACCP guidelines, which also states that mediastinal sampling or not is ‘a matter of judgment’ (4). Although ACCP guidelines suggest which tests should follow inconclusive tissue samplings (6), there are no recommendations on same-day order of invasive tests. The impact of bronchoscopy-induced coughing or bleeding on diagnostic yield of the next tissue sampling (e.g. x-ray-guided TTNAB) is unknown, but our results (mainly rural living; median age 69 years; no physician trained in the United States) suggest that three or more consecutive, invasive tests are both safe and efficacious (Tables 1 and 2). This is consistent with a UK study showing that EBUS is as safe in elderly (>70 years) as in younger patients (25). Thus, integrating diagnosis and staging in one session increases likelihood of following guidelines and shortens time delay but requires organisational factors that allow individualised workup and care (26).

A strength of our study is that none were lost to follow-up, resulting in unique data completeness. From our unit, we referred 14% (133 of the 937 patients) for surgery, equivalent to the Danish mean (27), and patients were diagnosed according to guidelines by specialists (23). The external validity of our study is high but only for patients referred for intended curative surgery for lung cancer, but not for the total lung cancer population, or the even larger population with suspected lung cancer. Thus, we cannot establish a true yield of same-day, invasive workup in lung cancer workup. On the contrary, our study included the patients who were most difficult to diagnose and stage (3, 6). These patients constitute a minority of the total lung cancer population, and need the highest number of both invasive and non-invasive tests to pass the ‘needle's eye’ of correct diagnosis (NSCLC), low stage, and sufficient performance and physiological reserves to recover after surgery (3, 4, 13). As most lung cancer patients present with metastatic disease (2, 28) where staging and diagnosis are often achieved with a single biopsy using the principle of the least invasive method (6), we believe that a final diagnosis and stage is achievable in a higher number of patients than reported here.

In the future, we will study the impact of same-day, invasive workup in unselected patients with suspected lung cancer, and compare it to multiple-day workup. Including data on patient-related outcomes and quality of life will elucidate patients’ preferences, and data on health-care expenses will challenge previous findings of costs being positively related to number of workup visits (29, 30).

In conclusion, we demonstrated that lung cancer workup with multiple invasive procedures in one day including a median of three different invasive techniques in one session is an efficacious and safe method resulting in valid results in patients with small tumour burden and localised disease eligible for surgical resection. Prospective studies should ascertain workup on an intention-to-treat basis, and possibly include patient-related outcomes systematically.
